# The impact of handwriting difficulties on compositional quality in children with developmental coordination disorder

**DOI:** 10.1177/0308022616650903

**Published:** 2016-06-16

**Authors:** Mellissa M Prunty, Anna L Barnett, Kate Wilmut, Mandy S Plumb

**Affiliations:** 1 Lecturer in Occupational Therapy, Brunel University London, UK; 2 Professor in Psychology, Oxford Brookes University, Oxford, UK; 3 Reader in Psychology, Oxford Brookes University, Oxford, UK; 4 Lecturer in Sport Science, Federation University, Victoria, Australia

**Keywords:** Development coordination disorder, DCD, handwriting, compositional quality, pausing, children

## Abstract

**Introduction:**

There is substantial evidence to support the relationship between transcription skills (handwriting and spelling) and compositional quality. For children with developmental coordination disorder, handwriting can be particularly challenging. While recent research has aimed to investigate their handwriting difficulties in more detail, the impact of transcription on their compositional quality has not previously been examined. The aim of this exploratory study was to examine compositional quality in children with developmental coordination disorder and to ascertain whether their transcription skills influence writing quality.

**Method:**

Twenty-eight children with developmental coordination disorder participated in the study, with 28 typically developing age and gender matched controls. The children completed the ‘free-writing’ task from the detailed assessment of speed of handwriting tool, which was evaluated for compositional quality using the Wechsler objective language dimensions.

**Results:**

The children with developmental coordination disorder performed significantly below their typically developing peers on five of the six Wechsler objective language dimensions items. They also had a higher percentage of misspelled words. Regression analyses indicated that the number of words produced per minute and the percentage of misspelled words explained 55% of the variance for compositional quality.

**Conclusion:**

The handwriting difficulties so commonly reported in children with developmental coordination disorder have wider repercussions for the quality of written composition.

## Introduction

The quality of written composition is vital to academic performance. Students need to be able to write good quality compositions, which require skills in areas such as idea generation, vocabulary use, organisation, spelling, grammar and punctuation. According to Olive (2004), writing is one of the most cognitively complex tasks that humans engage in. It involves the interaction of a wide range of cognitive processes all competing for limited working memory resources (Kellogg, 1996; McCutchen, 1996). In young typically developing children who are beginning to write, some of the first skills to be taught are lower level ‘transcription skills’ which consist of two components: spelling and handwriting/typing (Berninger and Swanson, 1994). In young writers, transcription skills are so demanding that they act as a constraint on the higher level processes of writing such as planning and revision. This has been demonstrated in many studies where handwriting speed (the number of letters produced in a timed alphabet-writing task) was found to correlate highly with text length and quality of the composition produced in time limited tasks (Berninger and Swanson, 1994; Berninger et al., 1992).

The importance of developing writing skills is emphasised in educational systems worldwide. For example, in the United Kingdom (UK) the 2013–2015 Key Stage 2 (8–11 years) English written examinations awarded 37 out of 40 points for writing ability and reserved only three points for handwriting quality (legibility) (Department for Education (DfE), 2013). It is assumed that by the end of Key Stage 2 children will have developed automaticity in the lower level transcription skills of handwriting and spelling. However, if a child has handwriting difficulties, there may be wider repercussions for the quality of their written composition. Children with developmental coordination disorder (DCD) are one group in particular known for a high incidence of difficulties with handwriting (Prunty et al., 2013, 2014; Rosenblum and Livneh-Zirinski, 2008). DCD is the term used to refer to children who present with motor coordination difficulties unexplained by a general medical condition, intellectual disability or neurological impairment (American Psychiatric Association (APA), 2013). Children with DCD face many barriers in performing everyday activities both at home and at school (Blank et al., 2012). However, difficulties with handwriting are perhaps the most significant and one of the main reasons for referral to occupational therapy (Miller et al., 2001; Rosenblum and Livneh-Zirinski, 2008; Prunty et al., 2013, 2014). Given the high incidence of handwriting difficulties reported in this population together with the link between handwriting speed and quality of written composition, it is surprising that very few studies have examined the quality of writing in children with DCD. Dewey and colleagues (Dewey et al., 2002) report the only study to examine writing quality in children with DCD in the English language. However, writing quality was only a small component of their investigation as the main focus was on examining factors such as attention, learning and psychosocial adjustment in children. Nevertheless, the children with DCD were reported to score more poorly than controls on subtests of the Woodcock–Johnson Psycho-Educational Battery – Revised (WJ-R) (Woodcock and Johnson, 1989). This is a very general educational assessment but did include an evaluation of punctuation and vocabulary, writing to dictation tasks, proofreading, writing fluency, punctuation, spelling and word usage. However, one of the barriers to interpreting the results of Dewey et al. (2002) is the lack of information on the exact nature of the writing tasks and how they were measured.

The relationship between handwriting difficulties and writing quality has yet to be considered in children with DCD. This is important for occupational therapists working with these children, as what may manifest as a handwriting difficulty may have wider implications for the child. Therefore, the purpose of this exploratory study was twofold: firstly to examine the compositional quality of writing in children with DCD and secondly to ascertain whether their transcription skills influence compositional quality. Measures of transcription, including the handwriting product, handwriting process, spelling ability and the percentage of spelling errors in the text, were used to enable a thorough evaluation of the relationship between transcription and compositional quality.

## Method

### Participants

Twenty-eight children with DCD between eight and 15 years old (27 boys, one girl) and 28 age (within four months) and gender matched typically developing (TD) controls were included in the study. All participants were of White British ethnicity.

#### DCD group

Children for the DCD group were recruited through advertising at parent support groups, schools and through the research group website. All children were assessed in line with European guidelines (Blank et al., 2012) and met the Diagnostic and Statistical Manual of Mental Disorders (DSM-5) diagnostic criteria for DCD (APA, 2013). The children had significant motor difficulties, with performance below the 10th percentile (24 below the 5th, 4 below the 10th) on the movement assessment battery for children 2nd edition test (MABC-2) (Henderson et al., 2007) (see Table 1), which examines motor competency. These motor difficulties had a significant impact on their activities of daily living, as reported by their parents and evident on the MABC-2 checklist (Henderson et al., 2007). The British picture vocabulary scale 2nd edition (BPVS-2) (Dunn et al., 1997) was used to give a measure of receptive vocabulary, which correlates highly with verbal IQ (Glenn and Cunningham, 2005). This was in at least the average range for all children, confirming the absence of a general intellectual impairment. No child had a formal diagnosis of ADHD. The children were also assessed on the reading and spelling components of the British Ability Scales Second Edition (BAS-II) (Elliot, 1996). No children had a formal diagnosis of dyslexia or other language impairment.

**Table 1.** Age and scores for developmental coordination disorder (DCD) and typically developing (TD) groups on selection measures.

	DCD *n* = 28			TD *n* = 28			
Selection Measures	Mean	SD	Range	Mean	SD	Range	*p*
Age in years	10.61	2.23	8.03–15.00	10.95	2.12	8.04–14.11	.441
MABC-2 test percentiles:							
Total test score	3.45	2.96	.10–9.0	43.37	25.4	16.0–95.0	<.001*
- Manual dexterity	6.41	8.12	.50–37.0	51.07	26.82	9.0–98.0	<.001*
- Aiming and catching	21.55	23.64	.50–84.0	64.67	20.41	25.0–98.0	<.001*
- Balance	5.98	4.67	.10–16.0	30.42	19.85	9.0–91.0	<.001*
BPVS-2 standard score	108.9	14.4	86–143	110	12.2	92–140	.655
BAS-II spelling standard score	95.8	13.7	68–126	111	12.7	89–132	<.001*
BAS-II reading standard score	109.5	13.8	82–137	122	12.6	93–142	<.001*

* *p* ≤ .050.

MABC-2: Movement Assessment Battery for Children test component; BPVS-2: British Picture Vocabulary Scale; BAS-II: British Ability Scales Second Edition.

#### TD control group

The control group was recruited through local primary and secondary schools in Oxfordshire, England. Teachers were asked to use their professional judgement to identify children without any motor, intellectual or reading/spelling difficulties. To ensure the children identified were free of these difficulties, they were individually tested using the MABC-2 (Henderson et al., 2007), BPVS-2 (Dunn et al., 1997) and the reading and spelling components of the BAS-II (Elliot, 1996). Children were included in the control group if they scored at least at the level expected for their age on all measures (standard score 85 or higher).

Children from both groups with a diagnosis of dyslexia, specific language impairment and/or those who had English as a second language were excluded from the study. Children in both groups who had a reported physical, sensory or neurological impairment were also excluded.

The study was approved by the University Research Ethics Committee at Brunel University London. Written consent was ascertained from the children’s parents and verbal assent was ascertained from the participants.

### Measures

#### The writing task

As part of a broader study, the Detailed Assessment of Speed of Handwriting (DASH) (Barnett et al., 2007) was chosen to provide a broad assessment of handwriting speed across a range of tasks. This includes a 10-minute ‘free-writing task’, considered more similar to classroom writing activities than the other shorter/copying tasks in the test. The task provides the opportunity to compose a piece of text about the topic of ‘my life’. Before commencing the task, a page is presented to the child containing different facets/topics of life. The child is reassured that the topics are only suggestions and that he/she can write about one topic or several, but should aim to write continuous text rather than produce a list (Barnett et al., 2007). They are given 1 minute to think of ideas to write about and make notes on the page if they wish. They were instructed to use their everyday handwriting. The DASH has UK norms for children aged nine to 16 years. The internal reliability of the total score for the DASH is between α  =  .83 to .89 and the inter-rater reliability for all four tasks is .99, as reported in the test manual.

#### Written composition

The Wechsler Objective Language Dimensions (WOLD) (Rust, 1996) criteria were used to evaluate the quality of the DASH free-writing scripts. The WOLD was chosen based on its close alignment with the English national curriculum grading system (DfE, 2013) for English and its ease of application to a variety of writing tasks. The six WOLD criteria are: ideas and development, organisation, vocabulary, sentence structure, grammar, and capitalisation and punctuation (Rust, 1996). Each one is scored on a scale from 1 to 4. Table 2 illustrates a score of 1 and 4 for each of the six criteria. The scores from each area are summed to form a total raw score. The groups were compared using the raw scores.

**Table 2.** The Wechsler Objective Language Dimensions scoring criteria for a score of ‘1’ and ‘4’ (taken from the test manual: Rust, 1996).

	Scoring criteria	
Item	Score of 1	Score of 4
Ideas and development	Weak ideas, minimally supported with little or no extension of details.	Extensive development of ideas. Uniqueness, interest to audience, strong support of main idea.
Organisation, unity and coherence	Lack of plan, incoherent.	Organised, smooth flow using transitions and sequences. No wandering from the theme/plan.
Vocabulary	Very simplistic, lacks variety. May be unclear or inappropriate.	Precise, appropriate, accurate. Imaginative and appealing to the reader.
Sentence structure and variety	Poor sentence structure. Many errors that inhibit fluency and clarity.	Excellent control and formation of sentences. Variety of structure and length. Few errors in structure.
Grammar and usage	Poor grammar and word usage, frequent errors.	No errors or very few in proportion to amount of text.
Capitalisation and punctuation	Frequent/serious errors that interfere with communication.	No errors or very few in proportion to amount of text, which do not interfere with clarity.

*Note*. This table only provides information on two ends of the scoring criteria. The full range was applied in the analyses.

In order to control for legibility bias or bias due to misspelled words, each script was typed up and misspelled words were corrected prior to analysing the quality of writing using the WOLD criteria. The first author, who was blind to group membership, initially scored all of the typed compositions. To check the reliability of scoring, 20 scripts (10 DCD, 10 TD) were randomly selected and scored by an external rater (a psychologist with particular expertise in children’s writing). The rater was blind to the nature of the study and group allocation of the scripts. The inter-rater reliability for the total raw score of the WOLD was .89.

#### Spelling errors

Spelling errors were recorded from the DASH free-writing scripts before typing them up. Illegible words were not included as misspelled words. The total number of misspelled words was summed for each participant and then calculated as a percentage of the number of legible words produced on the DASH free-writing task.

#### Additional measures for correlation and regression analyses

In our previous studies on handwriting performance in children with DCD, various aspects of handwriting were analysed and reported in detail (Prunty et al., 2013, 2014). The same measures were used in the regression analyses in the current study to ascertain their influence on the quality of written composition. These measures are described briefly below. For a fuller explanation see Prunty et al. (2013, 2014).

#### Handwriting product

The DASH (Barnett et al., 2007) was chosen to examine handwriting speed in our previous studies as it is the only standardised handwriting speed test with UK norms for nine to 16 year olds. In addition to the free-writing task described above, it also provides an opportunity to examine a range of other handwriting tasks (copying and writing from memory). The number of words per minute produced during the 10-minute free-writing task (previously reported in Prunty et al. (2013)) was used to examine the relationship between this measure and compositional quality.

#### Handwriting process

When completing the DASH tasks, the participants wrote with an inking pen on paper placed on a Wacom Intuos 4 digitizing writing tablet to record the movement of the pen during handwriting. The writing tablet transmits spatial and temporal data about the pen as it moves across the surface. Eye and Pen version 1 (EP1) software (Alamargot et al., 2006) was used to analyse the data and the following measures were examined.

*Execution speed* (cm/sec): This is the speed of the pen when it is in contact and moving on the page. This measure was used in the current study to examine the relationship between execution speed and compositional quality.

*Pausing during writing*: This is the percentage of time during the task where the pen was either off the page (in-air pause), or halted on the page (on paper pause). In Prunty et al. (2014) it was reported that the DCD group paused for a greater percentage of the task, had a tendency to pause for longer, with more pauses over 10 seconds, and also paused within words, which is an indication of lack of automaticity in writing (Prunty et al., 2014; Kandel et al., 2006). These measures were used in the current study to examine the relationship between pausing during writing and compositional quality.

### Data analysis

#### Group comparisons

Differences between the TD and DCD group (*n* = 28) were initially explored for each of the WOLD components illustrated in Table 3 as well as for the overall total raw scores. *T*-tests were used to investigate group differences for all components that were normally distributed. In cases where variables were not normally distributed, Mann–Whitney *U*-tests were conducted.

**Table 3.** A comparison of the mean scores using the Wechsler objective language dimensions (WOLD) scoring criteria for the developmental coordination disorder (DCD) and typically developing (TD) groups.

	DCD *n* = 28			TD *n* = 28		
WOLD Scores	Mean	SD	Range	Mean	SD	Range
Total WOLD raw score*	11.35	3.49	6–19	14.85	3.90	8–23
Ideas and development	2.14	.89	1–4	2.50	1.07	1–4
Organisation*	1.50	.63	1–3	2.03	.79	1–3
Vocabulary*	2.32	.90	1–4	2.89	.83	2–4
Sentence structure*	1.78	.73	1–3	2.25	.64	1–4
Grammar*	1.71	.71	1–3	2.42	.57	2–4
Capitalisation and punctuation*	1.89	.78	1–4	2.75	.84	1–4

Note. *p ≤ .050

### Correlation and regression analyses

#### Selection measures, spelling and compositional quality

Bi-variate correlations were conducted to examine relationships between the inclusion measures of age, spelling, reading, vocabulary and MABC-2 total, and manual dexterity scores and the WOLD raw scores. In addition, the percentage of spelling errors in the free-writing task was also examined. The correlations were conducted with each group separately; variables that were significantly related to writing quality in each group were then entered into a step-wise regression analysis to ascertain whether they had a predictive relationship with writing quality.

#### Handwriting product and process measures and compositional quality

In order to examine the relationship between writing quality and the handwriting product (words per minute) and process measures (execution speed on the free-writing task (cm/s), percentage of overall pausing on the DASH free-writing task, the percentage of time pausing over 10 seconds and percentage of within word pauses), bi-variate correlations were conducted with each group separately; variables that were significantly related to writing quality in each group were then entered into a step-wise regression analysis to ascertain whether any of the handwriting measures had a predictive relationship with writing quality.

## Results

### Group comparisons

#### Written composition

There was a significant effect of group (DCD < TD) for the total WOLD raw score *t*(54) = −3.53, *p* = .001, *d* = −0.47, and five out of the six analytical components of the WOLD including organisation (*U* = 246.0, *Z* = −2.57, *p* = .01, *d* = −0.34), vocabulary (*U* = 262.0, *Z* = −2.25, *p* = .024, *d* = −0.30), sentence structure (*U* = 260.5, *Z* = −2.37, *p* = .018, *d* = −0.31), grammar (*U* = 190.0, *Z* = −3.62, *p* < .001, *d* = −0.48) and, capitalisation and punctuation (*U* = 180.5, *Z* = −3.64, *p* < .001, *d* = −0.48). There was no effect of group for ideas and development (*U* = 317.0, *Z* = −1.28, *p* = .200, *d* = −0.17). The WOLD raw scores and analytical scores are presented in Table 3.

#### Spelling errors

The DCD group had a higher percentage of misspelled words in the DASH free-writing task (*Mdn* = 6.25) compared to the TD group (*Mdn* = 1.99), *U* = 197.0, *Z* = −3.19, *p* = .001, *d* = −0.42.

### Correlation analyses

#### Selection measures, spelling and compositional quality

For children with DCD, four of the measures (age, total and manual dexterity score of the MABC-2, percentage of misspelled words) were significantly correlated with the WOLD raw score. Age and spelling ability were significantly related to text quality in the TD group. Table 4 shows the Spearman correlations for the WOLD raw scores.

**Table 4.** Wechsler Objective Language Dimensions raw score correlations with selection measures and measures of the handwriting process.

	DCD *n* = 28	TD *n* = 28
Selection measures		
Age	.49**	.69**
% of spelling errors in the script	−.54**	−.62**
BAS-II spelling^a^	.30	−.03
BAS-II reading^a^	.31	.04
BPVS-2^a^	.25	.07
MABC-2 total^a^	.45*	−.04
MABC-2 manual dexterity^a^	.43*	−.09
Handwriting process measures		
Number of words per minute^a^	.58**	.63**
Overall pausing (%)	−.40*	−.46*
Pausing over 10 seconds (%)	−.18	−.55**
Pausing within words (%)	−.43*	.09
Execution speed of free-writing (cm/s)	.24	.33

Note. *p ≤ .050 **p ≤ .010^a^ standard score

DCD: developmental coordination disorder; TD: typically developing; BAS-II: British Ability Scale; BPVS-2: British Picture Vocabulary Scale; MABC-2: Movement Assessment Battery for Children test component.

#### Handwriting product and process measures and compositional quality

The results indicated a significantly positive relationship between the number of words produced per minute and the WOLD raw scores for both groups. A significantly negative relationship was found between the overall percentage of pausing and the WOLD raw scores for both groups. A significant negative relationship was found for percentage of pausing that occurred within words for the DCD group only. The percentage of pausing above 10 seconds was related to text quality for the TD group only. Table 4 presents correlations for the WOLD raw scores.

#### Regression analysis

The final stage of analysis used the results from the correlations above to determine which of the measures were predictive of the compositional quality of the writing produced by each of the groups. Separate regressions were conducted for each group as a result of the different patterns of correlations.

For children with DCD, the step-wise multiple regressions were conducted using the number of words per minute on the free-writing task, percentage of misspelled words, percentage of within word pausing and MABC-2 total standard score. Age was not included as it correlated too highly with the number of words per minute (*r* = .78). Since the number of words per minute has been shown to be a predictor of writing quality in the literature, this was included instead of age. In addition, the MABC-2 manual dexterity score was not included as it correlated too highly with the total test score (*r* = .93), indicating a problem with multicollinearity. The results of the regression indicated that two predictors explained 55% of the variance (*R*^2 ^= .58, *F*(2,25) = 17.38, *p* < .001). It was found that the number of words per minute significantly predicted writing quality (β = .497, *p* = .001), as did the percentage of misspelled words (β = −.494, *p* = .001). The other variables did not add to the amount of variance explained by these two measures.

For the TD group a step-wise multiple regression was conducted using the number of words per minute on the free-writing task, percentage of pausing above 10 seconds, and percentage of misspelled words. Age was not included as it correlated too highly with the number of words per minute. The results of the regression indicated that only one variable explained the most variance in the TD group. In the model, the number of words per minute explained 38% of the variance (*R*^2 ^= .40, *F*(1,26) = 17.50, *p* < .001).

## Discussion

The only previous study in the English language that examined writing ability in children with DCD used very general educational assessments rather than specific handwriting tests and focussed on co-occurring deficits in areas such as attention, reading, learning and psychosocial adjustment (Dewey et al., 2002). The current study examined the writing ability of children with DCD without other diagnoses in a more focussed manner by using more specific writing assessments. The results indicated that the DCD group performed significantly below their TD peers on all analytical items in the WOLD with the exception of ideas and development. In addition, their overall total score for writing quality was below their TD peers and they had a higher percentage of misspelled words, despite performing within the average range as a group in the BAS-II spelling test.

Examining writing quality using the WOLD scoring criteria, which are closely aligned to the national curriculum for England's grading system (DfE, 2013) for English and capture the main aspects of written composition, was a first step in terms of providing information on difficulties with writing in children with DCD. This study found that there were clear difficulties in areas such as sentence structure and grammar, which suggests that the DCD group had difficulties expressing their ideas within appropriately composed sentences. Previous studies examining the handwriting process have demonstrated that children with DCD pause for over 10 seconds at times during writing (Prunty et al., 2014), a pattern of behaviour which has, in adults, been associated with planning content (Alamargot et al., 2010). Although increased planning might be thought to be associated with better quality compositions, the excessive pausing for long periods reported by Prunty et al. (2014) was not associated with better quality of writing in the DCD group in the current study.

One reason for the poor written compositions within the DCD group could stem from the reduced amount of text produced, which gave less opportunity to develop the content. Indeed, regression analyses revealed that the number of words produced per minute explained a significantly large proportion of the variance in compositional quality, as did the percentage of misspelled words produced in the text. Our findings may suggest that the cognitive resources available for writing are consumed at the level of transcription in children with DCD and therefore there is a lack of resources available to dedicate to compositional quality.

The DCD group performed within the average range when formally tested on spelling ability, yet made a higher percentage of spelling errors during the writing task. While the spelling task involved writing single words under no time constraints, the free-writing task involved integrating and managing all the processes of writing. This may have placed excessive demand on working memory resources, therefore impacting on the process of retrieving spellings and the overall quality of writing. Whilst spelling and handwriting are both considered as transcription skills, surprisingly few studies have examined the nature of the relationship between the two. One study on children with dyslexia (Sumner et al., 2014) reported that handwriting skill was constrained by spelling ability, evident through excessive pausing within misspelled words and the emergence of spelling ability as a predictor of handwriting speed. These findings suggest a more complex link between spelling and handwriting than previously considered and the possibility that difficulties with handwriting impact on spelling performance, particularly in a task as demanding as free-writing. This again may be attributed to reduced working memory resources, where the demands of handwriting are so great in children with DCD that spelling performance, along with the higher level processes of writing, are negatively impacted. This is supported by findings from the current study, where spelling errors in the text, rather than single word spelling ability, were found to predict compositional quality. However, it is important to note that while this study measured some aspects of language and its impact on writing performance, the examination of other aspects of language skill (such as word retrieval or working memory) were outside the scope of this study. One limitation of this study is the ability to generalise the findings to children with DCD who have co-occurring disorders. This study controlled for factors such as reading ability, spelling ability, language and attention in order to understand handwriting difficulties in a sample of children with DCD. However, future research needs to consider children with co-occurring disorders given the constraints of language on handwriting production (Connelly et al., 2012; Sumner et al., 2014).

One of the strengths of using the WOLD scoring criteria in this study was its close alignment with England's national curriculum for English. This was appropriate from the perspective of DCD, as the European guidelines on assessment mention academic achievement and school productivity as areas affected by the disorder (Blank et al., 2012). Although academic performance is a complex factor to measure and was not the focus of the current study, there may be a link between handwriting dysfunction and academic achievement, at least within the English writing curriculum. In the current study we were not able to ascertain school grades for the Standard Assessment Tests (SATs) for English in the participants with DCD. This would have been interesting since the SATs marking criteria for English at the time of this study would have aligned with those from the WOLD. Further work is needed to investigate this area in greater detail in children with DCD.

## Conclusion

This study has shown that difficulties with transcription have real implications for the quality of text produced by children. The quality of the written composition is what is judged in the educational system, yet handwriting serves as the crucial medium through which it is produced. The clinical implications of this study relate not only to the importance of intervention but in the approaches that occupational therapists apply when addressing difficulties with handwriting. Therapists need to think beyond the motor aspects of handwriting skill and look at the broader aspects of writing, such as spelling and compositional skill. While it is apparent that children with DCD need support to acquire efficient skills in handwriting, further research needs to be undertaken to examine whether strategies specifically to enhance the quality of their compositional skills would be beneficial.

## Key messages

- Handwriting speed is a predictor of writing quality in children with DCD.
- Occupational therapists need to consider the impact of handwriting skill on broader aspects of writing performance.
- Interventions to increase handwriting skills in children with DCD may support their writing performance.

## What the study has added

This study is the first to examine the impact of handwriting difficulties on compositional quality in children with DCD. It supports the need for handwriting intervention in this group.
